# THE SWALLOW, A TARGET TO FOLLOW THE RESTAURATION OF CONSCIOUSNESS IN ACQUIRED BRAIN INJURY

**DOI:** 10.2340/jrm.v57.42692

**Published:** 2025-07-01

**Authors:** Anne CHARLOTTE LERICK, Eléonore SEQUEIRA, Jean GLENISSON, Virgil ROLLAND, Grégoire PRUM, Eric VERIN

**Affiliations:** 1 University Rouen Normandie, Normandie University, GRHVN UR 3830, Rouen; 2 Physical Medicine and Rehabilitation Center ‘‘les Herbiers’’, Bois Guillaume; 3 Rouen University Hospital, Rouen, Department of Neurosurgery Intensive Care Department, Rouen; 4 Rouen University Hospital, Department of Pulmonary Rehabilitation, Rouen, France

**Keywords:** dysphagia, swallowing disorders, consciousness, disorders of consciousness, traumatic brain injury

## Abstract

**Introduction:**

Brain injuries are the leading cause of disorders of consciousness and are often complicated by swallowing disorders. The aim of this study was to determine whether a correlation existed between swallowing and level of consciousness in patients with acquired brain injury.

**Methods:**

This pilot and observational study was conducted in the post intensive care coma arousal rehabilitation on 10 patients with acquired brain injury with disorder of consciousness and swallowing disorder evaluated with the Coma Recovery Scale–Revised (CRS-R) CRS-R evaluation or WHIM scale and a SWallowing Disorders in Disorders of Consciousness (SWADOC) assessment, both conducted in the same timeline frame. Swallowing function was assessed using the SWADOC scale. The level of consciousness was evaluated with the CRS-R and the Wessex Head Injury Matrix (WHIM). A Pearson correlation analysis was performed to examine the potential relationship between swallowing capacity and level of consciousness.

**Results:**

A strong correlation was identified between the CRS-R and WHIM scales with the SWADOC evaluation. Indeed, the correlation between SWADOC and CRS-R reached 0.70, while the correlation between SWADOC and WHIM was above 0.60.

**Conclusion:**

These findings highlight the importance of integrating swallowing evaluation within the multimodal assessment of consciousness recovery.

## LAY ABSTRACT

The authors demonstrated a significant association between improvements in the oral phase of swallowing and the recovery of consciousness in patients with disorders of consciousness, based on the following:

- A clinical study.
- A cohort of consecutive patients diagnosed with disorders of consciousness in unresponsive wakefulness state, minimally consciousness state, and emerging from minimally consciousness state.
- A positive correlation between oral phase function and the trajectory of consciousness recovery.

Swallowing disorders are a frequent consequence of brain injury, with an estimated prevalence ranging from 37% to 78% in the acute phase following a stroke (1). These impairments can affect all stages of swallowing – oral, pharyngeal, and oesophageal (2) – and are observed in both traumatic brain injury (TBI) and stroke patients (3). Videofluoroscopic evaluations often reveal delayed or absent swallowing reflexes, impaired lingual control, and reduced pharyngeal peristalsis in affected individuals (4).

In patients with severe brain injury, assessing swallowing function poses a clinical challenge, particularly when consciousness is impaired. While several bedside screening tools exist for this population, a recent development – the SWallowing Disorders in Disorders of Consciousness (SWADOC) scale – has been specifically designed to evaluate swallowing in patients with disorders of consciousness (DoC) (5). Notably, this tool enables assessment of both the oral and pharyngeal phases of swallowing. This is of particular interest because the oral phase requires cortical coordination, whereas the pharyngeal phase is mediated by the nucleus ambiguus in the brainstem. The latter acts as a central pattern generator, orchestrating the pharyngeal sequence via cranial nerves, while integrating afferent inputs from both the oral phase and cortical regions (6).

Brain injury is the primary cause of disorders of consciousness, which encompass a spectrum of conditions including coma, unresponsive wakefulness syndrome (UWS), and the minimally conscious state (MCS) – subdivided into MCS minus (MCS–) and MCS plus (MCS+) – as well as the emergence from MCS (EMCS) (7). Among these, UWS represents the most severe form. In clinical settings, consciousness levels are routinely assessed using validated behavioural scales such as the Coma Recovery Scale–Revised (CRS-R) (8) and the Wessex Head Injury Matrix (WHIM) (9). The CRS-R in particular is designed to detect signs of cortical activation through structured tasks. However, it does not include swallowing assessment, despite the fact that the oral phase of swallowing could serve as a potential indicator of cortical engagement.

The aim of our study was therefore to investigate whether the severity of swallowing disorders correlates with the degree of consciousness impairment in patients with brain injury. We also sought to determine whether the functionality of the oral phase is specifically associated with CRS-R and WHIM scores, which could imply underlying cortical activation.

## Methods

### Study design and participants

This pilot and observational study was conducted in the post intensive care coma arousal rehabilitation unit of Rouen University Hospital, from September 2023 to March 2024. The institutional ethics committee, in accordance with the World Medical Association Declaration of Helsinki, approved the study protocol (E2023-64). Oral consent was given by the family to use the data for research anonymously.

Inclusion criteria were patients with acquired brain injury with disorder of consciousness and swallowing disorder evaluated with the CRS-R evaluation or WHIM scale and a SWADOC assessment, both conducted in the same time frame. Exclusion criteria were pre-existing diseases affecting swallowing, whether neurological, central, peripheral, neuromuscular, or otolaryngological.

### Procedure and data collection

Assessments of the level of consciousness and deglutition were always conducted in stable conditions that optimized patients’ awareness: respecting sufficient time after caregiving activities, rehabilitation sessions, or medications that can cause fatigue. If a patient experienced infection, surgery, or any other treatable condition, their evaluations of consciousness and swallowing were excluded from analysis.

The presence of a tracheostomy tube was not an exclusion criterion.

### Evaluation of level of consciousness: CRS-R and WHIM

The CRS-R is a standardized neurobehavioural assessment scale designed to evaluate the level of consciousness of patients with disorders of consciousness, validated in French (10). CRS-R scoring is based on the presence or absence of specific behavioural reactions to standardized sensory stimuli to classify patients in UWS, MCS, and EMCS. The level of consciousness is determined by the presence of an item linked to MCS–, MCS+ or EMCS. If no item is linked to MCS or EMCS during the evaluation, the patient is classified as having UWS.

The Wessex Head Injury Matrix (WHIM) (9) was also used for neurological measurement of the patient’s level of consciousness. This scale is used in routine practice in our unit. Patients were considered to be in a vegetative state with a score between 1 and 15, minimally conscious with a score between 16 and 46. With a score between 47 and 62, patients with severe TBI were considered to be in emerging post-traumatic amnesia (PTA). The WHIM is composed of 62 behaviours, ordered according to a recovery sequence of motor, cognitive, and social abilities determined by the order of reappearance of these signs in awakened patients. It ends when 10 successive behaviours are not observed, and the latest behaviour observed corresponds to the WHIM score.

### Evaluation of swallowing disorders: SWADOC

The SWADOC (5, 11) is a quantitative and qualitative scale. It is composed of 56 items: 48 qualitative items and 8 quantitative items for the “SWADOC score” belonging to the quantitative part. SWADOC is assessed by a speech therapist. A timer, flashlight, spoon, a cotton swab soaked in sweet and cold solution, and 5 mL of coloured thickened texture (IDDSI3, moderately thick) are necessary for the assessment. The quantitative part of this scale is divided in 2: 1 for the oral phase and 1 for the pharyngeal phase. Each item is scored on a 4-level scale, ranging from Level 0 (worst) to Level 3 (best). Scores are assigned as follows: 0 for Level 0, 1 for Level 1, 2 for Level 2, and 3 for Level 3. The oral and pharyngeal phase scores are calculated by summing the scores of their respective items (O1 to O4 for the oral phase, and P1 to P4 for the pharyngeal phase). The total SWADOC score is the combined sum of both phases.

### Statistical analysis

The unit of evaluation was the “patient day”. Evaluations with missing data were excluded from the analyses. Consequently, only days with evaluation of SWADOC and CRS-R were used to evaluate the correlation between these 2 scales, and only days with evaluation of SWADOC and WHIM were used to evaluate the correlation between SWADOC and WHIM.

After describing the number of subjects and the number of evaluable days per subject, the following correlations were sought using Pearson correlation coefficients, on evaluations with no missing data for either SWADOC or CRS-R. The CRS-R was expressed on a scale from 0 to 1, by dividing the number of points observed by the theoretical maximum number of points for this subject. Then, we sought a correlation between the total SWADOC score and CRS-R, the oral SWADOC score and CRS-R, and the pharyngeal SWADOC score and CRS-R.

Similarly, for subjects with no missing data for either SWADOC or WHIM, the following Pearson correlations were estimated for the total SWADOC score and WHIM score, the oral SWADOC score and WHIM score, and the pharyngeal SWADOC score and WHIM score.

To take intra-patient correlation into account, confidence intervals for Pearson correlation coefficients were calculated using Wald’s method after Fisher’s z-transformation and calculation of the variance by Jackknife per patient, using Student’s law with *n*-2 degrees of freedom, where “*n*” is the number of patients. Subjects were multi-included, and each patient could have several scale evaluations at various times.

## Results

**Fig. 1:**
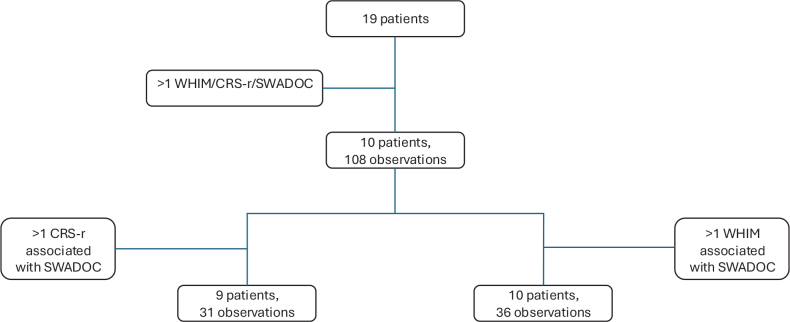
Flowchart resenting the number of patients included after meeting the inclusion criteria and exclusion criteria. Of 19 patients, 10 patients remained, with a mean ± SD of 10.80 ± 4.02 observations per patient. After exclusion of the missing SWADOC and CRS-R data, only 31 observations concerning 9 patients remained, with a mean ± SD of 3.44 ± 1.74 observations per patient (minimum = 1, maximum = 6). After exclusion of missing SWADOC and WHIM data, only 36 observations remained for 10 patients, with a mean ± SD of 3.60 ± 2.46 observations per patient (minimum = 1, maximum = 9).

During the study period, 188 evaluations were available (55 CRS-R, 72 WHIM, and 61 SWADOC) representing 108 evaluations, with a mean ± SD of 10.80 ± 4.02 evaluations per patient. After the exclusion of missing data, only 31 evaluations were available (3.44 ± 1.74 evaluations per patient; mean ± SD) in 10 patients, 7 men and 3 women, with a mean age of 33 ± 16 years (Fig. 1).

Clinical characteristics of these 10 patients are presented in Table I. The mean time between the injury and admission to the coma arousal unit was 64 ± 34 days. The mean time on ventilation was 25 ± 8 days. Six patients had a tracheotomy tube at the time of the first evaluation. Two of these patients had weaning of tracheotomy during the study period. At the first evaluation all patients were fed exclusively by gastrostomy, but 2 patients in EMCS were able to eat some oral food.

**Table I:** Medical data on the acute phase of acquired brain injury and general characteristics of patients

Sex, *n* (F, M)	3; 7
Age, years (m ± SD)	33 ± 16
Initial Glasgow Coma Scale (score)	5 ± 2
Number of days in intensive care unit (m ± SD)	53 ± 28
Number of days since the injury on inclusion	108 ± 73
Traumatic brain injury	5
Stroke	3
Anoxia	1
Infectious encephalopathy	1
Number of days under ventilation in ICU	25 ± 8
Number of days with a tracheotomy	122
Enteral nutrition (gastrostomy) on inclusion, *n*	10

The 4 oral phases (O1–O4) and the 4 pharyngeal phases (P1–P4) phases of SWADOC in relation to the number of patients in the different levels of consciousness (UWS, MCS–, MCS+, and EMCS) determined with the CRS-R, are presented in Table II. The efficacity of the pharyngeal phase maybe more impaired in UWS and MCS patients in comparison with MCS+ and EMCS, with a lack of initiation of the salivary swallowing reflex on command, and a longer latency of swallowing reflex triggering on oral stimulation.

**Table II:** Quantitative part of the SWADOC: in the first column items linked to oral or pharyngeal phase, and in the first line the different levels

Oral phase				
Item	Level 0	Level 1	Level 2	Level 3
1. Initiation of mouth opening	Mouth opening impossible or only with the therapist’s assistance	Mouth opening on lip stimulation	Mouth opening on presentation of spoon	Mouth opening on command (min 2/3)
	4/4 UWS	2/6 MCS+	1/12 EMCS	2/6 MCS+
	9/9 MCS–	2/12 EMCS		9/12 EMCS
	2/6 MCS+			
2. Endo-buccal secretions	Substantial amount of secretions (80–100%)	Moderate amounts of secretions (20–80%)	Few secretions (0–20%)	Moist mouth but without significant secretions
	1/9 MCS–	2/4 UWS	1/4 UWS	1/4 UWS
		7/9 MCS–	1/12 EMCS	1/9 MCS–
		1/6 MCS+		5/6 MCS+
		2/12 EMCS		9/12 EMCS
3. Lip prehension	No lip prehension (no reaction or tightening of the lips)	Incomplete lip prehension spontaneously or upon verbal stimulation	Appropriate lip prehension but not consistently or only upon verbal stimulation	Consistently correct and spontaneous lip prehension
	4/4 UWS	1/9 MCS–	1/6 MCS+	1/6 MCS+
	8/9 MCS–	2/6 MCS +	2/12 EMCS	7/12 EMCS
	2/6 MCS+	2/12 EMCS		
	1/12 EMCS			
4. Tongue propulsion	No tongue movement: passive movement of the bolus to the pharyngeal level, stagnation in mouth or expulsion when drooling	A few tongue movements but not sufficient to propel the bolus	Pathological tongue propulsion, possibly with post-swallowing stasis	Appropriate tongue propulsion
	1/4 UWS	2/4 UWS	1/4 UWS	3/6 MCS+
	7/9 MCS–	2/9 MCS–	1/6 MCS+	6/12 EMCS
	2/6 MCS +	1/12 EMCS	4/12 EMCS	
	1/12 EMCS			
Pharyngeal phase				
Item	Level 0	Level 1	Level 2	Level 3
1. Initiation of saliva swallowing reflex	No saliva swallowing spontaneously or upon stimulation	Saliva swallowing only upon stimulation	Saliva swallowing spontaneously and upon stimulation	Saliva swallowing upon command (min 2/3)
		3/9 MCS–	1/4 UWS	
	3/4 UWS	2/6 MCS+	1/9 MCS–	3/12 EMCS
	5/9 MCS–	4/12 EMCS	3/6 MCS+	
	1/6 MCS +		4/12 EMCS	
	1/12 EMCS			
2. Latency of swallowing reflex triggering upon stimulation	No triggering or cannot be completed	> 10 s	5 to 10 s	0 to 5 s
	3/4 UWS	1/9 MCS–		
	5/9 MCS–	3/12 EMCS	1/4 UWS	1/6 MCS+
	3/6 MCS+		3/9 MCS–	2/12 EMCS
			2/6 MCS+	
			7/12 EMCS	
3. Tracheotomy	Tracheotomy with inflated cuff	Tracheotomy with cuff ongoing deflation	Tracheotomy without cuff or with permanently deflated cuff	Tracheotomy with ongoing weaning, or no tracheotomy
	2/9 MCS–	2/9 MCS–	3/4 UWS	1/4 UWS
			4/9 MCS–	1/9 MCS–
			1/6 MCS+	5/6 MCS+
				12/12 EMCS
4. Bronchial congestion	Frequent bronchopneumonia or heavy congestion	Moderate congestion	Little congestion	No congestion
	2/9 MCS–	1/4 UWS	1/4 UWS	2/4 UWS
	1/6 MCS +	3/9 MCS–	1/9 MCS–	3/9 MCS–
		1/6 MCS+		4/6 MCS+

Four items are linked to the oral phase: (O1) Initiation of mouth opening, (O2) Endo-buccal secretions, (O3) Lip prehension, (O4) Tongue propulsion. Four items characterized the pharyngeal phase: (P1) Initiation of saliva swallowing reflex, (P2) Latency of swallowing reflex triggering upon stimulation, (P3) Presence/absence of tracheotomy, (P4) Bronchial congestion. Each subcategory is graduated into levels starting with Level 0 (worst) to Level 3 (best). For each subcategory of the oral (O1–O4) and pharyngeal (P1–P4) phases of the SWADOC the number of patients at the different levels of consciousness (UWS, MCS–, MCS+, and EMCS) determined with the CRS-R is noted.

**Fig. 2:**
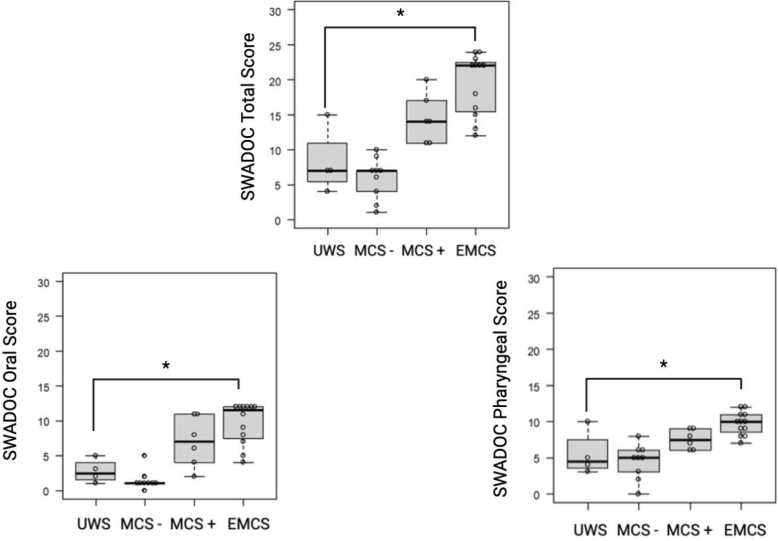
Box plots representing thanks to a Pearson correlation the relationship between level of consciousness separated into UWS, MCS–, MCS+, and EMCS (in the abscissa), and the total, oral, and pharyngeal score of the SWADOC (in the ordinates). Individual observations are represented by circles. The Pearson correlation coefficient between CRS-R and total SWADOC was estimated at 0.78 (95% CI 0.28 to 0.95, *p* = 0.008); between CRS-R and oral SWADOC it was estimated at 0.76 (95% CI 0.22 to 0.94, *p* = 0.01); between CRS-R and pharyngeal SWADOC it was estimated at 0.70 (95% CI 0.23 to 0.90, *p* = 0.009); between oral and pharyngeal SWADOC it was estimated at 0.76 (95% CI 0.44 to 0.91, *p* = 0.0005).

The Pearson correlation coefficient was 0.78 (95% CI 0.28 to 0.95, *p* = 0.008) for CRS-R (coded 0 = UWS, 1 = MCS-, 2 = MCS+, and 3 = EMCS) and total SWADOC, 0.76 (95% CI 0.22 to 0.94, *p* = 0.01) for CRS-R and oral SWADOC, and 0.70 (95% CI 0.23 to 0.90, *p* = 0.009) for CRS-R and pharyngeal SWADOC. Thus, the SWADOC scale was strongly correlated with the CRS-R scale (Fig. 2). The Pearson correlation coefficient was 0.71 (95% CI 0.33 to 0.89, *p* = 0.0009) for WHIM and total SWADOC, 0.67 (95% CI 0.20 to 0.89, *p* = 0.007) for WHIM and oral SWADOC, and 0.66 (95% CI 0.41 to 0.81, *p* < 0.0001) for WHIM and pharyngeal SWADOC. Thus, the SWADOC scale was strongly correlated with the WHIM scale.

## DISCUSSION

This is the first study, to our knowledge, to focus on the clinical correlation between swallowing and consciousness evaluations. The main finding of this study was a strong positive correlation between swallowing and level of consciousness according to CRS-R, WHIM, and SWADOC evaluations.

Until now, few studies have evaluated a correlation between swallowing and level of consciousness. The SWADOC was developed following Facial Oral Tract Therapy and the New Zealand Secretions Scale (NZSS) (5). In our study, the oral phase of the SWADOC was more impaired in the lower levels of consciousness. Indeed, patients with UWS and MCS– seemed to have less efficient mouth opening and lip prehension compared with higher levels of consciousness. Tongue propulsion also seemed to be more difficult for patients in MCS– than MCS+ or EMCS. Other studies also reported a less efficient oral phase in patients with UWS characterized by impaired lip prehension and impaired tongue propulsion responsible for post-swallowing oral stasis, in comparison with patients in MCS (5).

Like the oral phase, in our study, the pharyngeal phase of the SWADOC was also found to be more efficient at higher levels of consciousness. These results are not surprising, because patients need to manage their salivary secretions before weaning off tracheotomy. Our results are in line with those of others, who found more tracheotomy and more impairment of other components of the pharyngeal phase in patients at lower levels of consciousness (5). The mechanism involved is either an increase in the frequency of spontaneous swallowing or an improvement of pharyngeal propulsion with the enhancement of the level of consciousness (12). In our study, the pharyngeal phase was more impaired at lower levels of consciousness because of a lack of initiation of the salivary swallowing reflex on command, and a longer latency of swallowing reflex triggering on oral stimulation, suggesting a link between consciousness and swallowing reflex. This can be explained by the fact that the swallowing reflex is in a part a voluntary behaviour and may share cerebral regions responsible for consciousness.

The improvement in swallowing and oral phase of swallowing during disorders of consciousness may be a sign of cortical recovery. Electrophysiological studies, neuroimaging, and clinical observations showed the role of the cerebral cortex in the control of swallowing (13), contributing to both voluntary and involuntary aspects of swallowing through sensory processing and integration of stimuli from the mouth and throat, motor planning, and execution of swallowing movements, and providing feedback to brainstem swallowing centres. Therefore, it is possible that swallowing and consciousness share common cerebral regions responsible for voluntary tasks. Indeed, the primary sensorimotor cortex, premotor and supplementary motor areas, prefrontal cortex, cingulate cortex, insula, and thalamus are regions involved in the regulation of consciousness and volitional swallowing in a large network (14–16).

Nevertheless, this study has several limitations. Due to its observational, single-centre design, and small sample of patients, these results need to be considered with caution. Indeed, among 188 evaluations, only 31 SWADOC and CRS-R evaluations were available for 10 patients and only 36 SWADOC and WHIM evaluations. Some items like endo-buccal secretions, latency of swallowing reflex triggering, and bronchial congestion are sensitive to variations over time for test–retest reliability. Like others, we observed that these items depend partly on external factors (13), such as the time that last tracheostomy suction or mouth care, aerosol, or respiratory physiotherapy was performed.

Some clinical characteristics of our patients may have interfered with swallowing and level of consciousness evaluations. For the swallowing evaluation, some of our patients had severe oral apraxia, as reported by others (17), which may have altered the swallowing evaluation. Tracheostomy should also be discussed, as it could interfere with swallowing. This was absent for 40% of patients on inclusion and 2/6 patients were weaned off tracheostomy during the study. Nevertheless, the oral phase of SWADOC is not impacted by the presence or absence of tracheotomy. However, the presence of a tracheostomy must be considered as a possible cause in the lateness of swallowing reflex and may be a limitation of our study. Lastly, patients with disorders of consciousness are known to have an important fluctuation of wakefulness and awareness, which should induce variability in swallowing evaluations, thereby affecting both the internal consistency and test–retest reliability of the SWADOC (18). Although the results are represented in separated groups of level of consciousness, it must be underlined that the statistical analysis had to consider CRS-R as a linear scale to simplify the correlation analysis.

To conclude, our pilot study demonstrated a positive correlation between oral and pharyngeal swallowing and level of consciousness in patients with acquired brain injury. In fact, swallowing and consciousness seem to share some cortical regions, and their improvement could be a sign of cortical recovery. Nevertheless, our results need to be confirmed in longitudinal studies with larger samples, using behaviour scales and functional imaging to better understand the anatomical link between improvement in swallowing and level of consciousness.
